# The Anxiety of the 21st Century: The Paradox of Nomophobia. A Critical Examination of the Phenomenon in a Population with Eating Disorders

**DOI:** 10.1192/j.eurpsy.2025.478

**Published:** 2025-08-26

**Authors:** F. Marcolini, G. Buffa, B. Ferrari, D. De Ronchi, A. R. Atti

**Affiliations:** 1 Department of Biomedical and Neuromotor Sciences, University of Bologna, Bologna, Italy

## Abstract

**Introduction:**

In the present era, technology and medicine have become deeply intertwined, forming inseparable scientific disciplines. The mobile phone has become an omnipresent device, serving functions such as financial management, job organization, social networking, and internet access. While its benefits are undeniable, there is growing evidence of its potential pathological effects. One of the most recently identified issues is nomophobia—a term combining “no mobile,” “phone,” and “phobia,” which refers to the fear or anxiety of being without one’s mobile phone.

Nomophobia is considered a situational phobia of the contemporary era. Its symptoms include excessive reliance on mobile phones and pervasive anxiety about losing internet connectivity. Related conditions include “Ringxiety” and “phantom vibration syndrome,” where individuals perceive false notifications from their devices.

**Objectives:**

This study aims to assess the relationship between eating disorders (ED) and nomophobia. Additionally, it seeks to analyze the psychopathological components of nomophobia and evaluate the characteristics of the Nomophobia Questionnaire (NMP-Q), currently used for its assessment.

**Methods:**

A questionnaire was administered at the Study and Care Unit for ED in Bologna, Italy, between January 2023 and May 2024. Alongside tests evaluating the social and psychopathological characteristics of the outpatients (including the STAI-Y for anxiety assessment), the Italian version of the NMP-Q was employed.

**Results:**

The study included 104 patients (97 females and 7 males) with an average age of 21.8 years (range 18-44). The results showed that 100% of the subjects exhibited symptoms of nomophobia, with 21.3% displaying severe nomophobia. Among these, 94.4% tested positive for state anxiety, and 100% for clinically significant trait anxiety. This suggests that nomophobia may reflect not only current anxiety symptoms but also an anxious trait, indicating a predisposition to heightened reactivity and anxiety in response to environmental stimuli.

**Conclusions:**

The data highlight the alarming pervasiveness of internet addiction in contemporary society, with nomophobia being a significant manifestation. Given its substantial consequences, it is crucial to deepen our understanding of this condition and its underlying psychosocial determinants. This will enhance our knowledge and aid in developing more effective prevention strategies. However, a paradox arises: if nearly everyone is affected by nomophobia, it challenges the traditional definition of a disease. Further research into the NMP-Q test’s structure and specificity is necessary, as high prevalence rates may question its current measurement validity.

**Disclosure of Interest:**

None Declared

